# Single-Component Organic Solar Cells Based on Intramolecular Charge Transfer Photoabsorption

**DOI:** 10.3390/ma14051200

**Published:** 2021-03-04

**Authors:** Ken-ichi Nakayama, Tatsuya Okura, Yuki Okuda, Jun Matsui, Akito Masuhara, Tsukasa Yoshida, Matthew Schuette White, Cigdem Yumusak, Phillip Stadler, Markus Scharber, Niyazi Serdar Sariciftci

**Affiliations:** 1 Department of Material and Life Science, Graduate School of Engineering, Osaka University, Osaka 565-0871, Japan; yokuda@chem.eng.osaka-u.ac.jp; 2 Faculty of Engineering, Yamagata University, Yamagata 992-8510, Japan; twa10633@st.yamagata-u.ac.jp (T.O.); masuhara@yz.yamagata-u.ac.jp (A.M.); yoshidat@yz.yamagata-u.ac.jp (T.Y.); 3 Faculty of Science, Yamagata University, Yamagata 990-8560, Japan; jun_m@sci.kj.yamagata-u.ac.jp; 4 Department of Physics and Materials Science Program, University of Vermont, Burlington, VT 05405-0125, USA; mwhite25@uvm.edu; 5 Linz Institute of Organic Solar Cells (LIOS), Physical Chemistry, Johannes Kepler University, Linz 4040, Austria; Cigdem.Yumusak@jku.at (C.Y.); philipp.stadler@jku.at (P.S.); markus_clark.scharber@jku.at (M.S.); Serdar.Sariciftci@jku.at (N.S.S.)

**Keywords:** organic solar cell, intramolecular charge transfer, photon energy loss, exciton binding energy

## Abstract

Conjugated donor–acceptor molecules with intramolecular charge transfer absorption are employed for single-component organic solar cells. Among the five types of donor–acceptor molecules, the strong push–pull structure of DTDCPB resulted in solar cells with high *J*_SC_, an internal quantum efficiency exceeding 20%, and high *V*_OC_ exceeding 1 V with little photon energy loss around 0.7 eV. The exciton binding energy (EBE), which is a key factor in enhancing the photocurrent in the single-component device, was determined by quantum chemical calculation. The relationship between the photoexcited state and the device performance suggests that the strong internal charge transfer is effective for reducing the EBE. Furthermore, molecular packing in the film is shown to influence photogeneration in the film bulk.

## 1. Introduction

Organic photovoltaic devices typically employ a bulk heterojunction (BHJ) structure, where donor and acceptor molecules are microscopically mixed. This system has proven highly successful, and the power conversion efficiency of organic photovoltaic devices has exceeded 16% [1], with near-unity internal quantum efficiency and high fill factor (FF). However, photon energy loss at the donor–acceptor (D–A) interface remains problematic as it reduces the photovoltage and ultimately limits the power conversion efficiency. The success of the BHJ system is based on efficient photogeneration at the donor–acceptor interface taking advantage of the lowest unoccupied molecular orbital (LUMO) offset energy. This relaxation process promotes photogeneration but also causes substantial photon energy loss. In typical BHJ systems the lost energy is 1.2 eV for P3HT:PC61BM and 0.93 eV for PTB7:PC71BM, which indicates that more than 50% of the absorbed photon energy is wasted. Recently, the reduction and optimization of the LUMO offset in the BHJ system has been studied and reported [2,3].

In contrast, silicon solar cells do not have this problem because photogeneration occurs in the film bulk immediately after photon absorption. This situation is explained by the small exciton binding energy (EBE), which is defined as the energy difference between the photoexcited state and the free carrier (transport) state. The silicon crystal has high dielectric constant and small effective mass, resulting in a small EBE [4]. In contrast, the EBE in organic films is significantly larger, which is attributed to the low dielectric constant of organic materials that cannot screen the Coulomb attraction between the bound electron/hole pair [5]. Promoting photodissociation in the film bulk of single-component organic semiconductors remains challenging and could provide a promising pathway toward higher photovoltage in organic photovoltaics.

In the BHJ system, the absorbed photon produces Frenkel excitons in the film bulk which diffuse to the D–A interface and produce charge transfer (CT) excitons driven by the LUMO offset energy. The photodissociation process from the CT state to free carriers has been extensively studied. It has been reported that this CT exciton at the D–A interface is easily dissociated into free carriers with almost no energy barrier [6]. Therefore, if a photoexcited state, such as a CT exciton, can be produced directly from the absorbed photons, then photogeneration from the film bulk would be expected with no photon energy loss from the LUMO offset at the D–A interface.

In this study, we investigated the possibility of single-component OPV devices taking advantage of the spontaneous photodissociation from the CT state. To achieve photogeneration in the single-component film, it is important to design the photoexcited state to enhance the CT character. Therefore, we focus on several molecules that exhibit an intramolecular charge transfer photoexcited state. These molecules are normally composed of distinct donor and acceptor units.

Thus far, donor–acceptor linked molecules by an inert linker have been extensively studied from the viewpoint of extending the lifetime of a charge-separated state mainly in dilute solution [7,8,9]. There are several reports of single-component systems based on polymers having donor and acceptor units [10,11,12]. However, in such a linked system, the dissociated charges are relaxed in a secondary process following photoexcitation, resulting in photon energy loss in the same manner as in the BHJ system. There have been several reports on the single-component OPVs employing D–A conjugated polymers [13,14]. For the intramolecular CT molecules, the highest occupied molecular orbital (HOMO) and LUMO are overlapped, and the photoexcited state is delocalized between the donor and acceptor unit. In this case, the charges forming a CT state are expected to be separated from the delocalized photoexcited state to the surrounding molecules of the same type with smaller energetic barrier. This mechanism is analogous to bulk photogeneration in silicon solar cells. We investigate single-component devices using several intramolecular CT molecules and the relationship between the device performance and photoexcited state through theoretical prediction of the EBE using a quantum chemical calculation. We identify a molecule exhibiting efficient charge generation upon photoexcitation. The presented results suggest that molecules with a strong CT-state are promising absorbers in organic solar cells and detectors.

## 2. Materials and Methods

**Figure 1:**
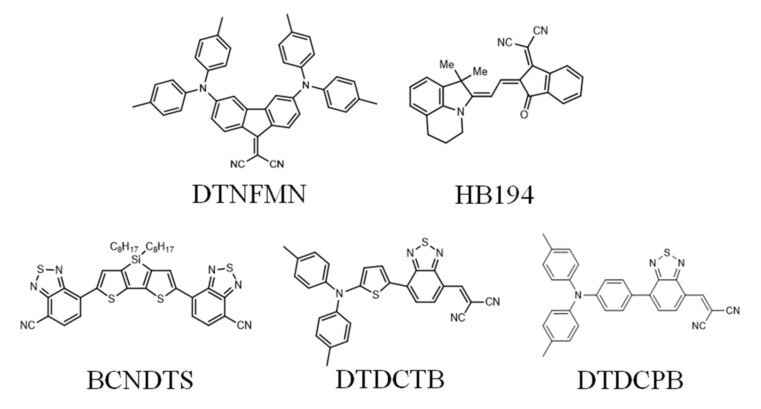
Chemical structure of intramolecular charge transfer (CT) molecules used in this study.

The chemical structures of the CT compounds used in this study are shown in Figure 1. Each molecule contains donor and acceptor units within one molecule. These molecules are reported as donor materials in the BHJ system prepared by vacuum evaporation. 2-[(7-{4-[N,N-Bis(4-methylphenyl)amino]phenyl}-2,1,3-benzothiadiazol-4-yl)methylene]propanedinitrile (DTDCPB) and 2-{[7-(5-N,N-Ditolylaminothiophen-2-yl)-2,1,3-benzothiadiazol-4-yl]methylene}malononitrile (DTDCTB) have triphenylamine unit as a donor and benzothiadiazole unit as an acceptor [15,16]. 2-((Z)-2-((E)-2-(1,1-Dimethyl-5,6-dihydro-4H-pyrrolo[3,2,1-ij] 3 uinoline-2(1H)-ylidene)ethylidene)-3-oxo-2,3-dihydro-1H-inden-1-ylidene)malononitrile (HB194) is a type of merocyanine dye [17]. 2-(3,6-Bis(di-p-tolylamino)-9H-fluoren-9-ylidene)malononitrile (DTNFMN) has a rigid p-conjugated structure [18]. 7,7’-(4,4-bis-(n-octyl)dithieno[3,2-b:2’,3’-d]silole-2,6-diyl) dibenzo[c][1,2,5]thiadiazole-4-carbonitrile (BCNDTS) was designed as an A-A-D-A-A-type electron donor [19]. DTDCTB and DTDCPB are linear-shaped D-A molecules, while DTNFMN and BCNDTS are D-A-D and A-A-D-A-A type molecules, respectively. These materials were purchased from Lumtec Corp. (New Taipei City, Taiwan) and were used without further purification. An ITO-coated glass substrate was cleaned using a cleaning solution (Semico Clean 56, Furuuchi Chemical, Tokyo, Japan), water, acetone, and finally isopropyl alcohol by ultrasonication for 10 min in each case. After UV/O_3_ treatment for 20 min, PEDOT:PSS (Clevios™ AI4083, Heraeus, Hanau, Germany) was spin-coated onto the cleaned ITO surface, and the resulting substrate was baked at 120 °C under vacuum for 10 min. The single-component active layer was deposited by vacuum evaporation. Finally, an Al cathode was evaporated. The deposition rate was typically 0.2 nm/s for the organic layer and 1 nm/s for the Al electrode.

The device was encapsulated with a glass lid and then tested in air. Current density–voltage (*J–V*) curves were measured using a source measure unit (Model 2400, Keithley, Solon, OH, USA). The photovoltaic performance was measured under AM 1.5G illumination with an intensity of 100 mW cm^−2^ using a solar simulator system (CEP-2000, BUNKOUKEIKI, Hachiohji, Japan). Incident photon-to-current conversion efficiency (IPCE) spectra under monochromatic incident light were measured using the same system. The illumination intensity dependence of the device performance was measured by using a variable light intensity solar simulator (MOS-100, BUNKOUKEIKI, Hachiohji, Japan). Absorption spectra and the optical energy gap of the prepared film were measured using a UV/vis spectrophotometer (V-570, JASCO, Hachiohji, Japan). The energy level of the HOMO was determined using photoelectron yield spectroscopy (PYS-201, Sumitomo Heavy Industries, Tokyo, Japan), and the energy level of the LUMO was estimated by subtracting the optical energy gap from the HOMO level.

## 3. Results

### 3.1. Film Characterization

**Figure 2:**
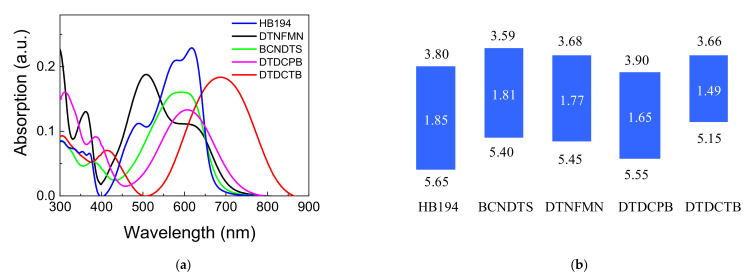
(**a**) UV-vis absorption spectra of the vacuum-deposited films of each molecule, (**b**) HOMO and LUMO energy levels and energy gap in eV.

The absorption spectra of the vacuum-deposited films of each molecule are shown in Figure 2a. Each film had a broad absorption band in the visible region. Particularly, DTDCTB showed a longer wavelength region, up to 850 nm. The HOMO–LUMO levels of these films are exhibited in Figure 2b. HB194 and DTDCPB have relatively deeper HOMO and LUMO levels. The thiophene unit in DTDCPB pushes up the HOMO level, resulting in the narrowest HOMO–LUMO gap among these compounds.

### 3.2. Device Performance Comparison

**Figure 3:**
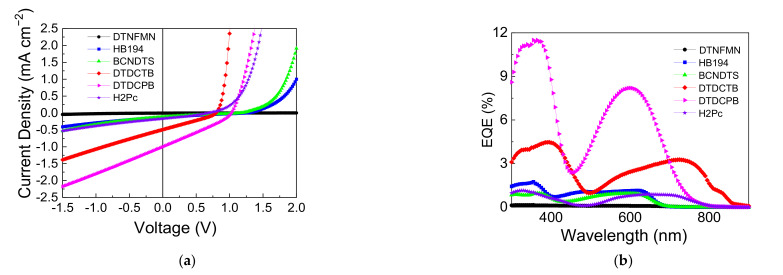
(**a**) *J*–*V* curves of the single-component photovoltaic cells under dark and AM1.5G illuminated conditions. (**b**) External quantum efficiency (EQE) spectra.

The *J–V* characteristics and the EQE of the single-component devices are shown in Figure 3. The performance parameters are summarized in Table 1. Each single-component device using the CT molecules showed good rectification behavior in dark conditions. We discussed the electric property of each material from the dark *J*–*V* curve for forward bias because it should reflect transport properties of the materials in the single-component system. Among these materials, DTDCPB showed the best photovoltaic performance metrics with *V*_OC_ exceeding 1 V and *J*_SC_ of approximately 1 mA/cm^2^. DTDCTB has a wide absorption band up to 850 nm and exhibited the second largest *J*_SC_. HB194 and BCNDTS showed high *V*_OC_, exceeding 1.2 V, but *J*_SC_ remained with small values. DTNFMN showed a very small photoresponse, which is attributed to the poor electrical properties. The internal quantum efficiency (IQE) was calculated according to the literature assuming double absorption paths and perfect reflection at the Al electrode [20]. The IQE for the DTDCPB device was 20% for the lower energy absorption band centered at 600 nm and up to 35% at the valley of absorption (465 nm). Therefore, DTDCPB is the most promising of the studied material for the single-component organic photovoltaic device. Among these compounds, linear D–A type molecules tend to show better performance than D–A–D type molecules. As a reference, phthalocyanine (H_2_Pc), which is a standard organic semiconductor and non-CT molecule, was investigated. CuPc has a wide absorption band, up to 800 nm, and good electrical property; nevertheless, it showed low PCE, of approximately 0.03%. It has been reported that PTB7, which is a standard donor polymer for the BHJ system, also shows very low performance in the single-component device [13].

The photon energy loss was estimated by subtracting the *V*_OC_ from the photoabsorption band edge energy. The photon energy loss for the five materials was low compared to conventional organic photovoltaic devices, approximately 0.7 eV, and their values were consistent despite the different performances. Although the device performance is much lower compared to the BHJ system, the magnitude of the photon energy loss has one of the lowest levels in the organic photovoltaic device. In contrast, H_2_Pc showed larger photon energy loss, as high as 0.94 eV, which corresponds to 60% of the absorbed photon energy.

In these systems, the photocurrent increases with increasing reverse bias. The dark current for the reverse bias remains with a small value for a large reverse bias; therefore, the net photocurrent can be estimated by subtracting the dark current from photocurrent, even for high reverse bias. The photocurrent in the DTDCPB device reached 6 mA/cm^2^ at −7 V, corresponding to approximately 60% of the EQE. This increase is interpreted as field-assisted photogeneration, which suggests that the geminate recombination process is responsible for the small *J*_SC_. We believe that the magnitude of photocurrent at reverse bias is a good indicator of potential as a single-component system.

**Figure 4:**
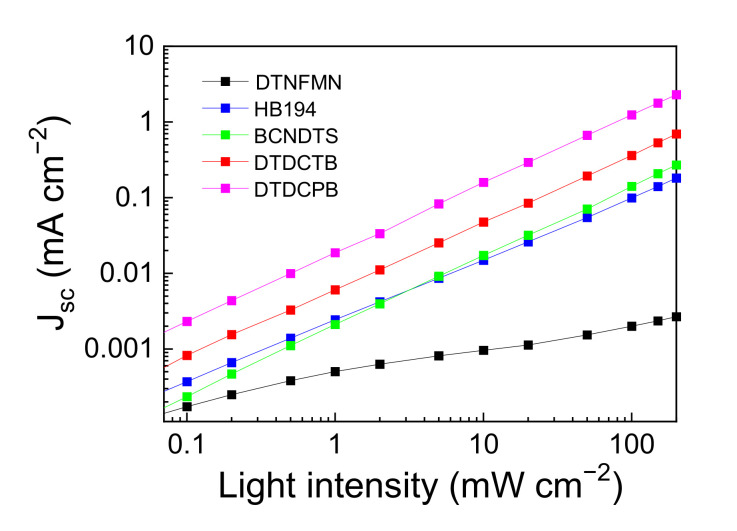
Light intensity dependence of *J*_SC_ for the single-component devices.

Figure 4 shows the light intensity dependence of the *J*_SC_ and *V*_OC_ for each device. The slope of *J*_SC_ is almost unity except for DTNFMN for a wide intensity range, from 0.1 to 200 mW/cm^2^. The relationship between *J*_SC_ and light intensity *I* is described as *J*_SC_ ∝ *I*^α^, where α = 1 corresponds to the absence of bimolecular recombination and α = 1/2 corresponds to perfect bimolecular recombination. The estimated α for DTDCPB and DTDCTB were 0.94 and 0.91, respectively, rivaling typical BHJ device performances. Generally, the high α close to unity in the BHJ system is explained by its segregated structure; holes and electrons conducted via the separate physical domains toward each electrode; therefore, they do not recombine. These values being close to unity suggest that even in the single-component system, bimolecular recombination is not dominant. The low performance is primarily attributed to the geminate recombination process before photodissociation as expected for the single-component organic system.

### 3.3. Exciton Binding Energy (EBE)

**Figure 5:**
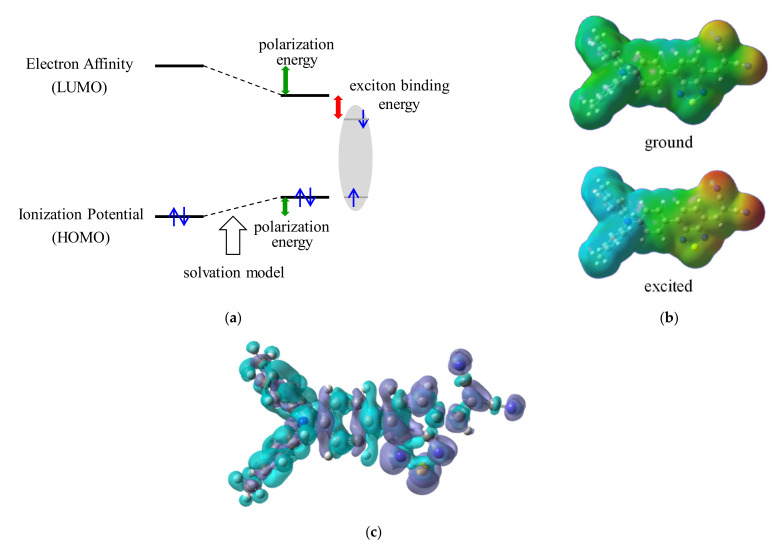
(**a**) Calculation scheme for exciton binding energy in the solid state. (**b**) Electrostatic potential distribution of the DTDCPB molecule. (**c**) Differential electron density distribution of the DTDCPB molecule upon photoexciation.

The low performance of the single-component device is attributed to a large EBE. Generally, EBE in an organic solid is larger than that in the inorganic crystal owing to its low dielectric constant. Here, we predicted the EBE for each molecule using quantum chemical solvation model and DFT calculations [21,22]. The calculation scheme is displayed in Figure 5a. The EBE is defined as the energetic difference between the photoexcited and free carrier states. The energy level of the free carrier state is called the transport level. The transport levels are stabilized by polarization of surrounding molecules in the solid state. In this scheme, this effect is simulated by using the solvation model. The dielectric constant as a solvent is calculated using Clausius–Mosotti’s relation from the calculated molecular volume and polarizability. Furthermore, the charge transfer distance upon photoexcitation Δ*r* and the HOMO–LUMO overlap parameter *S* in the singlet-excited state were estimated by using the multiwavefunction analyzer published by Liu [23].

The calculated physical parameters are summarized in Table 2. Ionization potential (IP), electron affinity (EA), energy gap (*E*_g_), excitation energy (*E*_x_), and EBE are indicated for the solvation model, that is, solid-state prediction. The IP in the solid state showed a good correlation with the experimental values estimated from photoelectron yield spectroscopy for the vacuum deposited films. The calculated EBE in the solid state with subtraction of *E*_x_ from *E*_g_ showed a negative value for DTDCPB and BCNDTS because the *E*_x_ corresponds to the absorption peak, whereas the real EBE is estimated from absorption band edge. Therefore, these values should be considered as relative values. DTDCPB, which had the highest performance, exhibited the smallest EBE, whereas HB194 and DTNFMN, with low performance, showed relatively high values. One of the important factors in this scheme is the estimated dielectric constant calculated from molecular polarizability and molecular volume. The high dielectric constant as a solvent stabilizes the transport levels; consequently, the EBE, that is, the energy difference between the excited state and transport levels, becomes smaller. Although the photovoltaic performance is affected by various factors, including charge transport properties, contact with the electrode, and recombination probability, we believe that the calculated EBE can partially predict the single-component performance.

The electronic state of DTDCPB upon photoexcitation is visualized based on the DFT calculation. The electrostatic potential distribution on the ground and excited states and the differential electron density upon photoexcitation are shown in Figure 5b,c, respectively. The DTDCPB molecule has a donor component of triphenylamine and an acceptor component of benzothiadiazole unit and cyano groups. On the ground state, the donor part is weakly polarized positively, and the acceptor part is weakly polarized negatively, resulting in a large dipole moment with 11 Debye. On the photoexcited state, this polarization is enhanced by intramolecular charge transfer. The differential electron density clearly indicates that an electron is transferred from the donor moiety to the acceptor moiety against the dipole upon photoexcitation. This polarized excited state with a strong CT character brought about a higher probability of photodissociation in the film bulk. To quantify the CT characters of the excited state, we focus on the absolute value of the photoinduced charge transfer distance Δ*r* and HOMO–LUMO overlap parameter *S*. Generally, photoexcitation with Δ*r* > 0.2 nm is called charge transfer absorption. DTDCPB showed the longest charge transfer distance, as long as 0.649 nm. In contrast, the compounds showing low performance, such as HB194 and DTNFMN, showed a smaller distance. From these results, Δ*r* is also important for the performance in the single-component system. *S* is related to the overlap integral between HOMO and LUMO. Higher *S* is desirable for charge transfer probability; however, separating the donor and acceptor part renders *S* smaller. Therefore, these parameters are in a trade-off relationship.

As mentioned in the introduction, the key point of this system is that the intramolecular CT state is directly produced by photoexcitation, and the excited state is conjugated through the donor and acceptor part. This situation is different from the conventional D–A linked system with an inert linker. In the single-component system based on the conjugated D–A molecules, it is important to enhance both Δ*r* and *S* simultaneously.

### 3.4. Effects of Film Structure in the DTDCPB Device

**Figure 6:**
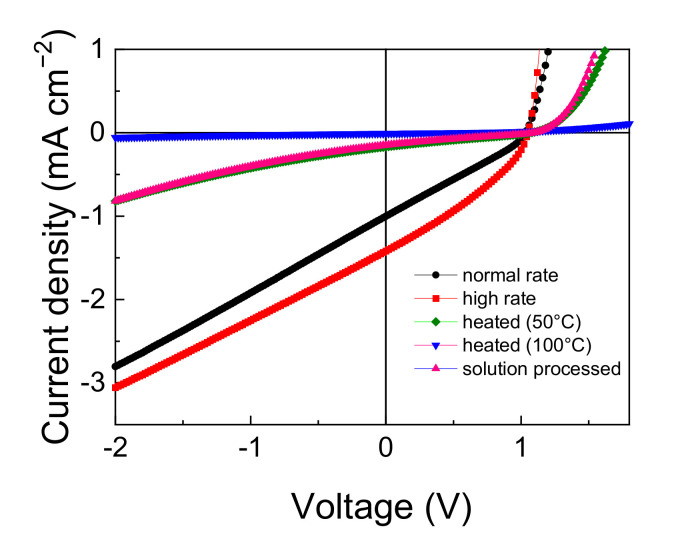
*J*-*V* curves of the DTDCPB single-component cells with various film preparation conditions under AM1.5G illumination.

The dissociation of CT excitons into free carriers within the bulk is expected to depend on the molecular packing in the solid state. The effect of film structure or molecular arrangement on the device performance was investigated for DTDCPB, which showed the highest performance. Several devices under different conditions were prepared, the *J–V* curves are displayed in Figure 6, and the performance parameters are summarized in Table 3. The deposition rate of the DTDCPB vacuum-deposited film was changed from the normal deposition rate of 0.2 nm/s to a higher deposition rate of 2 nm/s. This significantly improved the performance of the DTDCPB single-component device. *J*_SC_ increased 1.4 times, and even the FF was improved to 0.33. The vacuum-evaporated film of DTDPCB has a nearly amorphous structure, as observed by XRD. Therefore, it is difficult to discuss this difference based on film structure, but it suggests that the higher rate produces a molecular packing more suitable for the single-component device.

The effect of substrate heating during evaporation was investigated. The performance decreased with increasing temperature. This behavior is opposite to expectations for the effect on carrier transport. Generally, a slower deposition rate or substrate heating results in a more ordered structure and higher carrier mobility. The superior electrical properties lead to not only better carrier extraction but also better photodissociation because the Onsager–Braun model describing photodissociation probability includes carrier mobility. However, the photocurrent remarkably decreased in the more ordered films. These results suggest that the ordered film is disadvantageous for the photogeneration process, potentially due to a sub-optimal intermolecular dipole alignment in the crystalline bulk.

Finally, we prepared a solution-processed device by spin-coating DTDCPB solution in chloroform. However, the solution-processed device showed much lower performance, especially low *J*_SC_. There is also no information on the film structure from X-ray diffraction for the spin-coated film, but molecules tend to aggregate in the solution-processed film compared to vacuum-deposited films. These results and the effects of deposition rate, substrate temperature, and solution process device suggest that the film structure strongly affects the performance in the DTDCPB single-component device.

**Figure 7:**
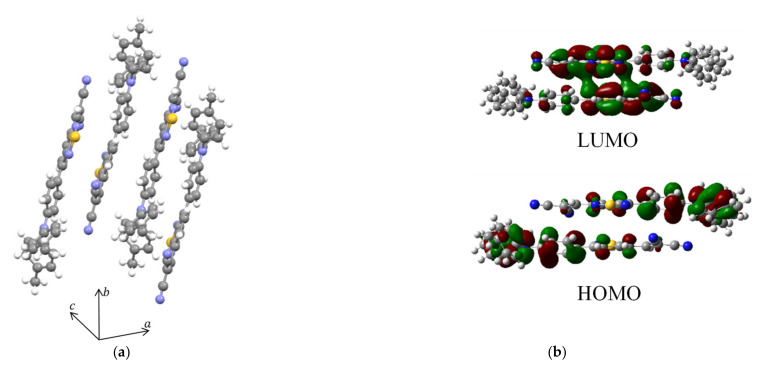
(**a**) Single crystal structure of DTDCPB. (**b**) Distribution of HOMO and LUMO for the DTDCPB dimer.

The above results, higher performance for higher deposition rate or deposition without substrate heating, can be explained by dimer formation in the solid-state film. A linearly linked D-A molecule, such as DTDCPB, necessarily has a large dipole moment. A strong Coulomb interaction between two dipole moments of the molecules would tend to form an antiparallel pair in the solid state. Indeed, the single-crystal structure of DTDCPB suggests antiparallel packing (Figure 7a). To clarify the impact of dimer formation on the excited state, we performed the same calculation of EBE, Δ*r*, and *S* for the DTDCPB antiparallel dimer taken out from the single-crystal structure (Figure 7b). The photoinduced charge transfer distance is largely canceled for the antiparallel dimer, resulting in a relatively larger EBE. This insight agrees with the obtained experimental results in the previous section. This dimer structure cannot be detected by X-ray diffraction; however, the film preparation conditions such as slow evaporation rate, high substrate temperature, and solution process generally brings about thermally more stable and ordered film structure. Thus, we inferred that these conditions should promote the formation of the dimer structure, resulting in a decrease of photovoltaic performance, mainly *J*_SC_. These results also support the concept that CT character in the excited state is important for photodissociation in the single-component film.

From these considerations, we propose the guiding principles for improving the performance of single-component organic solar cells. First, a molecular design that makes the charge transfer distance larger is important to reduce the EBE. Second, molecular design and film preparation conditions should be engineered to inhibit the antiparallel dimer. The absolute performance is still low in these proof-of-concept solar cells, yet these principles would help further improvement in the single-component system.

## 4. Conclusions

Several materials with intramolecular charge transfer absorption were utilized as single-component organic solar cells. With a strong push–pull structure, DTDCPB showed the highest performance as a single-component device. The electronic state calculation for the solid state suggests that these intramolecular CT molecules have lower EBE, which corresponds to the photovoltaic performance. The film structure also affects the performance, as antiparallel dimer formation in ordered films can reduce the efficiency of generation of free carriers in the bulk. The FF is still low presumably due to geminate recombination. To improve the performance in these materials, reducing the exciton binding energy is essentially required. For that purpose, enhancing the CT characters of the photoexcited state and increase of dielectric constant are important strategies. These insights suggest that further development of materials for single-component organic solar cells could promote photogeneration in the film bulk with a small photon energy loss, pushing organic photovoltaics closer to the Shockley-Queisser theoretical efficiency limit [24].

## Figures and Tables

**Table 1:** Device performance parameters of the single-component photovoltaic devices.

Material	*J*_SC_	*V*_OC_	FF	PCE	Photon Energy Loss
	(mA/cm^2^)	(V)	(%)	(%)	(V)
DTNFMN	0.00	1.05	15.5	0.00	0.72
HB194	0.12	1.25	22.6	0.04	0.60
BCNDTS	0.16	1.22	23.7	0.05	0.59
DTDCTB	0.50	0.79	28.8	0.11	0.70
DTDCPB	1.00	1.03	29.1	0.30	0.69
H_2_Pc	0.16	0.67	31.0	0.03	0.94

**Table 2:** Calculated exciton binding energy and related parameters in solid state based on the solvation model.

Compounds	IP (eV)	EA (eV)	*E*_g_ (eV)	*E*_x_ (eV)	EBE (eV)	Δ*r* (ang)	*S*
DTDCPB	−5.524	−3.438	2.086	2.292	−0.206	6.177	0.301
DTDCTB	−5.499	−3.400	2.099	1.980	0.119	4.125	0.372
DTNFMN	−5.554	−2.837	2.717	2.425	0.292	2.694	0.378
HB194	−5.901	−2.800	3.101	2.561	0.540	2.505	0.307
BCNDTS	−5.746	−3.562	2.184	2.216	−0.032	0.201	0.476

**Table 3:** Device performance parameters of the DTDCPB single-component cells with various film preparation conditions.

Device	*J*_SC_	*V*_OC_	FF	PCE
	(mA/cm^2^)	(V)	(%)	(%)
Normal rate	1.00	1.03	29.1	0.30
High rate	1.42	1.05	33.2	0.49
Heated at 50 °C	0.17	1.07	24.5	0.04
Heated at 100 °C	0.01	0.56	25.6	0.00
Spin-coated	0.15	1.09	23.1	0.04

## Data Availability

Data sharing not applicable.
